# Supplementary material for: EnzML: multi-label prediction of enzyme classes using InterPro signatures
Source: BMC Bioinformatics. 2012 Apr 25;13:61. doi: 10.1186/1471-2105-13-61 (PMC3483700; doi:10.1186/1471-2105-13-61)
Supplement: Addtional file 5 — The Java code to format the data files, evaluate and predict. The file enzml_java_code.tar.gz contains the Java code used to format database data to ARFF and XML formats, to execute cross and train-test (jackknife) evaluations and to record evaluation results to database. More information is included in the readme.txt file and the Javadoc files. The code can be used with a MySQL database. To use a different database software, other JDBC drivers might be required. [file 1471-2105-13-61-S5.gz › java_code/enzml2011/doc/uk/ac/ed/inf/csb/taweka/mulan/xml/MulanXml.html]

MulanXml


---


|  |  |  |
| --- | --- | --- |
| **Overview** \| **Package** \| **Class** \| **Use** \| **Tree** \| **Deprecated** \| **Index** \| **Help** | |  |
| **PREV CLASS**   NEXT CLASS | **FRAMES**    **NO FRAMES**     **All Classes** |  |
| SUMMARY: NESTED \| FIELD \| CONSTR \| METHOD | DETAIL: FIELD \| CONSTR \| METHOD |  |


---


## uk.ac.ed.inf.csb.taweka.mulan.xml Class MulanXml

```
java.lang.Object
  uk.ac.ed.inf.csb.taweka.mulan.xml.MulanXml
```

---

``` public class MulanXml extends java.lang.Object ```

Generates an XML file for labels in the Mulan format
http://mulan.sourceforge.net/ http://mlkd.csd.auth.gr/multilabel.html

Example of Mulan XML format for labels:

```
 labels xmlns="http://mulan.sourceforge.net/labels"> 
 <label name="label1">  
 	<label name="label12"></label>  
 	<label name="label13"></label>  
 	<label name="label14"></label>  
 	<label name="label15"></label> 
 </label>
 /labels>
```

**Version:**
:   30 Apr 2010

**Author:**
:   Luna De Ferrari luna.deferrari-at-ed.ac.uk

---

| **Field Summary** | |
| --- | --- |
| `static java.lang.String` | `LABEL_NAME_ATTRIBUTE`             the label tag name attribute |
| `static java.lang.String` | `LABEL_XML_TAG`             the label tag |
| `MulanLabel` | `m_root`             the xml tree root |
| `static java.lang.String` | `MULAN_XML_ROOT_TAG` |
| `static java.lang.String` | `MULAN_XML_ROOT_TAG_ATTRIBUTE_NAME` |
| `static java.lang.String` | `MULAN_XML_ROOT_TAG_ATTRIBUTE_VALUE` |


**Constructor Summary**

`MulanXml()`


| **Method Summary** | |
| --- | --- |
| `MulanLabel` | `findNode(java.lang.String label)`             Find a node by label |
| `MulanLabel` | `getRoot()` |
| `java.lang.String` | `toString()`             public MulanLabel removeNode(String label) { // find node MulanLabel node = this.findNode(label); // get parent MulanLabel parent = node. |

**Methods inherited from class java.lang.Object**

`equals, getClass, hashCode, notify, notifyAll, wait, wait, wait`

**Field Detail**

### LABEL\_NAME\_ATTRIBUTE

```
public static final java.lang.String LABEL_NAME_ATTRIBUTE
```

:   the label tag name attribute

    **See Also:**: Constant Field Values

---


### LABEL\_XML\_TAG

```
public static final java.lang.String LABEL_XML_TAG
```

:   the label tag

    **See Also:**: Constant Field Values

---


### MULAN\_XML\_ROOT\_TAG

```
public static final java.lang.String MULAN_XML_ROOT_TAG
```

**See Also:**: Constant Field Values

---


### MULAN\_XML\_ROOT\_TAG\_ATTRIBUTE\_NAME

```
public static final java.lang.String MULAN_XML_ROOT_TAG_ATTRIBUTE_NAME
```

**See Also:**: Constant Field Values

---


### MULAN\_XML\_ROOT\_TAG\_ATTRIBUTE\_VALUE

```
public static final java.lang.String MULAN_XML_ROOT_TAG_ATTRIBUTE_VALUE
```

**See Also:**: Constant Field Values

---


### m\_root

```
public MulanLabel m_root
```

:   the xml tree root


**Constructor Detail**

### MulanXml

```
public MulanXml()
```


**Method Detail**

### findNode

```
public MulanLabel findNode(java.lang.String label)
```

:   Find a node by label

    :   **Parameters:**: `label` - the node label (the name attribute value), such as 'abc' in ``` <label name="abc"></label> ``` **Returns:**: the node with that label, or null if not found

---


### getRoot

```
public MulanLabel getRoot()
```

---


### toString

```
public java.lang.String toString()
```

:   public MulanLabel removeNode(String label) { // find node MulanLabel node
    = this.findNode(label); // get parent MulanLabel parent = node. // remove
    child node }

    :   **Overrides:**: `toString` in class `java.lang.Object`


---


|  |  |  |
| --- | --- | --- |
| **Overview** \| **Package** \| **Class** \| **Use** \| **Tree** \| **Deprecated** \| **Index** \| **Help** | |  |
| **PREV CLASS**   NEXT CLASS | **FRAMES**    **NO FRAMES**     **All Classes** |  |
| SUMMARY: NESTED \| FIELD \| CONSTR \| METHOD | DETAIL: FIELD \| CONSTR \| METHOD |  |


---
